# *De novo* Transcriptome Generation and Annotation for Two Korean Endemic Land Snails, *Aegista chejuensis* and *Aegista quelpartensis*, Using Illumina Paired-End Sequencing Technology

**DOI:** 10.3390/ijms17030379

**Published:** 2016-03-15

**Authors:** Se Won Kang, Bharat Bhusan Patnaik, Hee-Ju Hwang, So Young Park, Tae Hun Wang, Eun Bi Park, Jong Min Chung, Dae Kwon Song, Hongray Howrelia Patnaik, Jae Bong Lee, Changmu Kim, Soonok Kim, Hong Seog Park, Jun Sang Lee, Yeon Soo Han, Yong Seok Lee

**Affiliations:** 1 Department of Life Science and Biotechnology, College of Natural Sciences, Soonchunhyang University, 22 Soonchunhyangro, Shinchang-myeon, Asan, Chungcheongnam-do 31538, Korea; bioksw@naver.com (S.W.K.); drbharatbhusan4@gmail.com (B.B.P.); hwamux@naver.com (H.-J.H.); cindysory@naver.com (S.Y.P.); wth14@naver.com (T.H.W.); eunbi9154@naver.com (E.B.P.); jong6922@daum.net (J.M.C.); elegangce@naver.com (D.K.S.); hhowrelia.patnaik@gmail.com (H.H.P.); 2 Trident School of Biotech Sciences, Trident Academy of Creative Technology (TACT), Chandaka Industrial Estate, Chandrasekharpur, Bhubaneswar, Odisha 751024, India; 3 Korea Zoonosis Research Institute (KOZRI), Chonbuk National University, 820-120 Hana-ro, Iksan, Jeollabuk-do 54528, Korea; jblee@jbnu.ac.kr; 4 National Institute of Biological Resources, 42, Hwangyeong-ro, Seo-gu, Incheon 22689, Korea; snubull@korea.kr (C.K.); sokim90@korea.kr (S.K.); 5 Research Institute, GnC BIO Co., LTD. 621-6 Banseok-dong, Yuseong-gu, Daejeon 34069, Korea; 5022daniel@gmail.com; 6 Institute of Environmental Research, Kangwon National University, 1 Kangwondaehak-gil, Chuncheon-si, Gangwon-do 243341, Korea; sljun@kangwon.ac.kr; 7 College of Agriculture and Life Science, Chonnam National University, 77 Yongbong-ro, Buk-gu, Gwangju 61186, Korea; hanys@chonnam.ac.kr

**Keywords:** Bradybaenidae, land snails, transcriptome, *de novo* assembly, simple sequence repeats

## Abstract

*Aegista chejuensis* and *Aegista quelpartensis* (Family-Bradybaenidae) are endemic to Korea, and are considered vulnerable due to declines in their population. The limited genetic resources for these species restricts the ability to prioritize conservation efforts. We sequenced the transcriptomes of these species using Illumina paired-end technology. Approximately 257 and 240 million reads were obtained and assembled into 198,531 and 230,497 unigenes for *A. chejuensis* and *A. quelpartensis*, respectively. The average and N50 unigene lengths were 735.4 and 1073 bp, respectively, for *A. chejuensis*, and 705.6 and 1001 bp, respectively, for *A. quelpartensis*. In total, 68,484 (34.5%) and 77,745 (33.73%) unigenes for *A. chejuensis* and *A. quelpartensis*, respectively, were annotated to databases. Gene Ontology terms were assigned to 23,778 (11.98%) and 26,396 (11.45) unigenes, for *A. chejuensis* and *A. quelpartensis*, respectively, while 5050 and 5838 unigenes were mapped to 117 and 124 pathways in the Kyoto Encyclopedia of Genes and Genomes database. In addition, we identified and annotated 9542 and 10,395 putative simple sequence repeats (SSRs) in unigenes from *A. chejuensis* and *A. quelpartensis*, respectively. We designed a list of PCR primers flanking the putative SSR regions. These microsatellites may be utilized for future phylogenetics and conservation initiatives.

## 1. Introduction

Mollusca represents the second most speciose phyla of all animals that inhabit marine, freshwater, and terrestrial habitats. Molluscs are highly diverse ecologically, and include economic aquaculture species, environmental biomarker species, destructive pests, and disease vectors. Eighty percent of mollusc species belong to the class Gastropoda, which is one of the most successful animal groups on earth. Gastropods live in a wide range of habitats: marine, freshwater, inland aquatic (including salt lakes), and terrestrial ecosystems. Terrestrial molluscs have a number of striking adaptations in their physiology, sense organs, reproduction, and development, which allow them to occupy a diverse range of niches. In fact, land snails are recognized as model systems for investigating shell morphological evolution as a result of adaptation to particular habitats [1,2], as well as reproductive and defensive behaviors [3].

The land gastropods of the family Bradybaenidae are distributed across a wide range of habitats in East Asia. This taxonomic family comprises land snails, terrestrial pulmonate gastropod molluscs, which are medium to small in size and belong to the superfamily Helicoidea. Based on karyotyping, one study documented the conservation of chromosome number in 36 species in the family Bradybaenidae [4]. The Bradybaenid snails of East Asia that belong to the genus *Aegista* are very closely related, and include 75 species and 112 subspecies [5]. The subgenera *Aegista* and *Plectotropis* are distributed mainly in Japan, China, and the Korean mainland [6], while the subgenera *Coelorus* and *Neoaegista* are endemic to Japan [7,8]. The Bradybaenidae snails of Korea have been classified into 24 species. Jeju Island contains a small but interesting assortment of these snails, including eight species out of which three belong to the genus *Aegista* [9]. The *Aegista* species identified on Jeju Island include *Aegista chejuensis*, *Aegista chosenica* [10], and *Aegista quelpartensis*. *A. chejuensis* and *A. quelpartensis* have been found restricted to Jeju Island with no reports from any other parts of the country. Aegista species of land snails are recorded from lowland broadleaf and evergreen forests, and are usually found under fallen trees or leaf litter or attached to the stems and leaves of plants [2].

*A. chejuensis* and *A. quelpartensis,* reported from under shrubs and stone piles on Jeju Island, are listed as Korean endemic species in the Korean Red List of Threatened Species, 2014. Moreover, *A. chejuensis* is classified as a vulnerable species owing to a decline in its numbers over recent years, due mainly to predation by natural enemies and habitat destruction as a result of forest development. With no regional conservation measures, these endemic species face the prospect of extinction in their natural habitat. Given the vulnerable status of this species, it is critical to identify genetic markers that can be used for phylogenetics and genetic mapping. Currently, genomic information for these species is scarce (for example, there are no NCBI entries), which limits our understanding of their phylogeography and our ability to identify immediate conservation priorities.

Next-generation sequencing (NGS) technologies have been used to sequence the transcriptomes of non-model organisms, thus providing genetic resources for use in functional genomics, phylogeographic/phylogenetic research, and conservation genomics [11,12]. A 454/Roche NGS platform produces long read lengths, while the Solexa/Illumina NGS platform produces shorter reads which are useful in downstream applications such as sequence annotation [13,14]. Most recent transcriptomic analyses conducted for molluscan species have used the Illumina HiSeq platform, owing to its efficiency and relatively low cost [15,16,17]. For instance, among snails, the *de novo* transcriptome of the pond snail *Radix balthica* has been studied using the Illumina platform [14]. A large-scale transcriptomic dataset for the freshwater snail *Oncomelania hupensis*, which acts as the intermediate host of *Schistosoma japonicum*, was also created with the Illumina sequencer [18]. Similarly, this technology was used to study transcriptome-wide expression analysis in four populations of the marine snail *Tegula atra* along the Chilean coast [19]. Despite the progress for marine and freshwater snails, transcriptomics research in land snails has been limited, with the exception of the Illumina transcript libraries derived from central nervous system, hepatopancreas, and foot muscle of the terrestrial snail pest *Theba pisana* [20]. The partial genomes of two land snail species from family Bradybaenidae, *Aegista diversifamilia* and *Dolicheulota formosensis,* have yielded a large amount of genetic data which may facilitate research on the evolutionary processes in gastropods [21].

In this study, we investigated the whole-body transcriptomes of the Korean endemic land snails *A. chejuensis* and *A. quelpartensis* using the Illumina NGS platform. We provide new genomic sequence information for these species and discuss its application in molecular taxonomy, functional genomics, and conservation genetics study.

## 2. Results and Discussion

### 2.1. Illumina Sequence Analysis and Assembly

**Figure 1:**
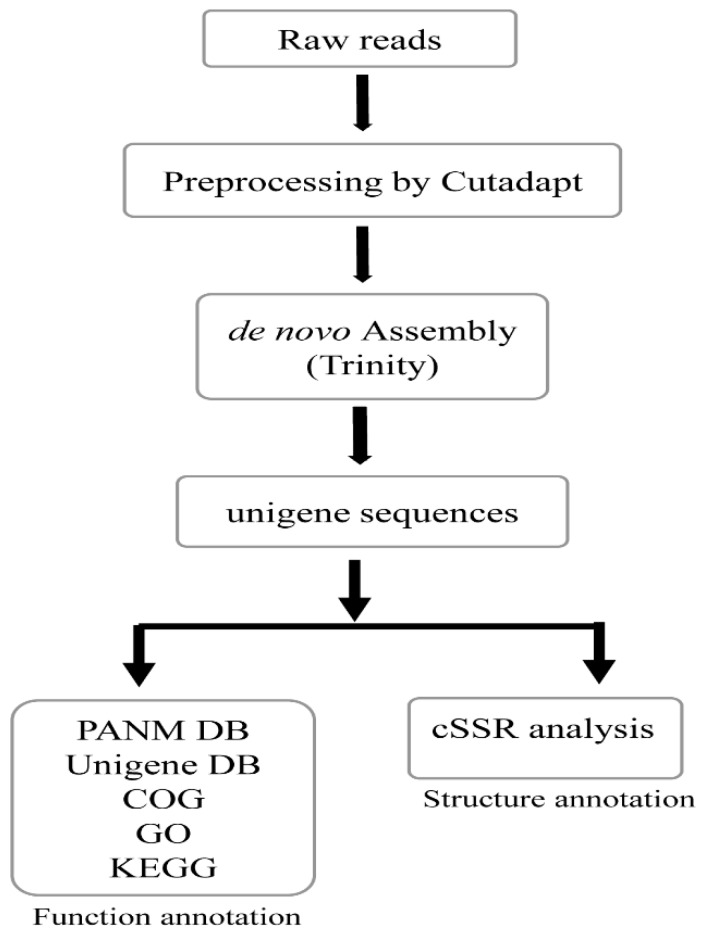
Schematic work-flow of the transcriptome analysis employed in the present study to annotate the unigenes of Korean endemic land snails, *Aegista chejuensis* and *Aegista quelpartensis*.

We obtained the whole transcriptomes of the Bradybaenidae land snails *A. chejuensis* and *A. quelpartensis* using the Illumina HiSeq 2500 sequencing platform. The transcriptome assembly and analysis workflow is shown in Figure 1. Each sequencing lane generated 2 × 50-nt independent reads from either end of a cDNA fragment. We obtained 256,655,870 (32,338,639,620 nt) and 239,242,058 (30,144,499,308 nt) raw reads for *A. chejuensis* and *A. quelpartensis*, respectively. After pre-processing the raw reads which included trimming adapter sequences, we recovered 99.83% and 99.79% of the sequencing reads for the *A. chejuensis* and *A. quelpartensis* transcriptomes, respectively (Table S1). A total of 253,220,985 (31,397,895,789 nt) and 235,525,993 (29,188,212,057 nt) clean reads remained, with average fragment lengths of 124 and 123.9 bp for *A. chejuensis* and *A. quelpartensis*, respectively. An overview of the transcriptome sequencing, assembling, and clustering results is presented in Table 1.

**Figure 2:**
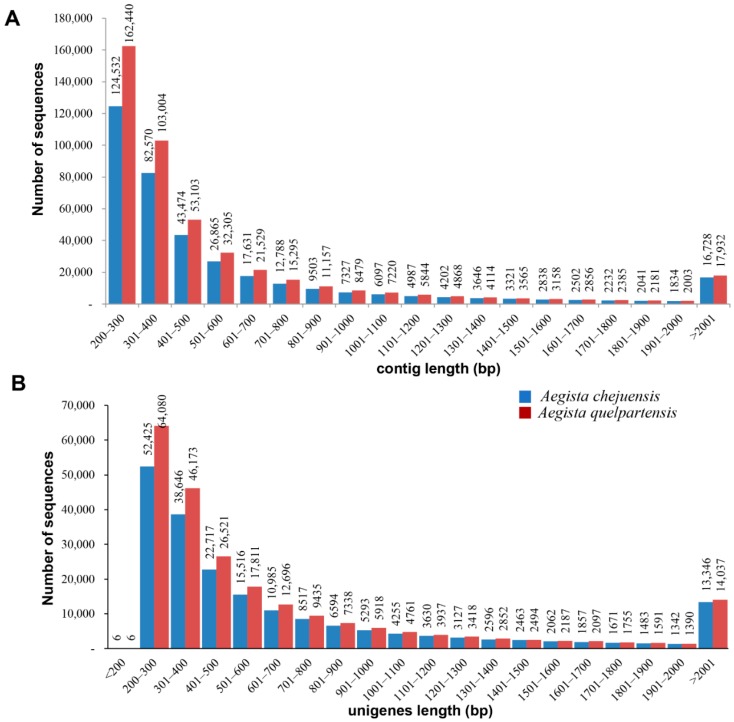
Size distribution of contigs (**A**) and unigenes (**B**) after assembly and clustering of the clean reads obtained from transcriptome sequencing of *A. chejuensis* and *A. quelpartensis*.

Clean reads were assembled using a Trinity *de novo* program (default sequence length: >200 nt) for contiguous, overlapping sequences (contigs). A total of 375,118 (229,108,084 nt) and 463,438 (269,776,350 nt) contig sequences were assembled for *A. chejuensis* and *A. quelpartensis*, respectively. The mean length and the N50 length of contigs in *A. chejuensis* were 610.8 and 788 bp, whereas in *A. quelpartensis* they were 582.1 and 719 bp, respectively. The longest contigs were 34,543 and 26,467 bp for *A. chejuensis* and *A. quelpartensis*, respectively. A size distribution analysis of the contigs in *A. chejuensis* (Figure 2A) revealed 74,114 contigs (19.8%) ranging from 501 to 1000 bp, 33,700 contigs (8.98%) ranging from 1001 to 2000 bp, and 16,728 contigs (4.46%) over 2001 bp in length. *A. quelpartensis* had approximately 88,765 contigs (19.15%) ranging from 501 to 1000 bp, 38,194 contigs (8.24%) ranging from 1001 to 2000 bp, and 17,932 contigs (3.87%) over 2001 bp in length. The previously reported Japanese scallop (*Mizuhopecten yessoensis*) transcriptome, which was created using the Illumina sequencing platform, generated contigs ranging from 100 to 29,088 bp in length, with an average of 436 bp [22]. In this study, we obtained a longer average contig length, with the longest in *A. chejuensis* being 34,543 bp. The average contig length in our assembly exceeded those of the non-model snail species *R. balthica* (536 bp average contig length) [14], the blood cockle *Anadara trapezia* (505 bp) [23], and the *Chlamys farreri* mantle transcriptome (249 bp) [24]. Generally, larger values for contig number, N50 length, average contig length, and maximum contig length are associated with superior assembly performance (although there are some exceptions) [25,26]. We calculated N50 by adding long contigs to short contigs until the summed length exceeded 50% of the total length of all contigs. The Trinity *de novo* assembler used in this study has been shown to outperform other top assemblers and is widely used across a variety of taxa [27,28,29,30].

Next, the unigenes (sequences not being extended on either side) were obtained by mapping the paired-end reads to contigs and using TGICL to form a single set of non-redundant unigenes. TGICL effectively removes redundancy and retains long, high-quality transcripts which are essential parameters for obtaining rich genetic information [31,32,33]. The analysis yielded 198,531 (145,998,300-nt) unigene sequences with average and N50 lengths of 735.4 and 1073 bp, respectively, for *A. chejuensis*. For *A. quelpartensis*, the clustering assembly yielded 230,497 unigene sequences with average and N50 lengths of 705.6 and 1001 bp, respectively. Of the assembled unigenes for *A. chejuensis*, 84,737 (42.68%) were longer than 500 bp, and 50,428 (25.4%) were longer than 1000 bp. In case of *A. quelpartensis*, 93,717 (40.66%) unigenes were longer than 500 bp, and 40,519 (17.58%) were longer than 1000 bp. The size distribution of assembled unigenes is shown in Figure 2B. The average length and N50 length of unigenes obtained in this study is greater than average length (453 bp) and N50 length (492 bp) of unigenes in the comprehensive transcriptome dataset for *Echinolittorina* snails [15]. Overall, based on the comparison of transcriptomes, we find a more effective *de novo* assembly in *A. quelpartensis* as it generated a larger number of transcripts from fewer raw read sequences.

### 2.2. Sequence Annotation and Homology Characteristics

The assembled unigene sequences of *A. chejuensis* and *A. quelpartensis* were aligned with protein and nucleotide databases using BLASTX and BLASTN analysis, respectively, at an *E*-value cutoff of ≤1.0 × 10^−5^. Table 2 reports the sequence-based annotation profiles of unigenes obtained against protein databases such as Protostome DB (PANM-DB), Clusters of Orthologous Groups of proteins (COG), Gene Ontology (GO), Kyoto Encyclopedia of Genes and Genomes (KEGG), and nucleotide database in Unigene DB. While PANM, Unigene, and COG DB were used as sequence annotation databases, GO, KEGG, and InterProScan analysis were used for enrichment analysis. The results indicated that, out of 198,531 and 230,497 unigenes for *A. chejuensis* and *A. quelpartensis*, a total of 68,484 (34.5%) and 77,745 (33.73%) unigenes, respectively, were annotated to the public databases. However, 65%–66% of unigenes were not annotated based on BLAST searches with protein sequences from the public databases. Most of these unigenes were relatively short sequences and, understandably, may lack conserved protein motifs and domains. Moreover, in addition to protein-coding genes, the transcriptome may also contain incompletely spliced introns, orphaned untranslated regions (UTRs), non-coding genes, and random transcriptional noise. Consequently, these are valid sequences, even though they were not annotated as proteins. Out of the public databases used for sequence annotation in this study, PANM-DB recorded the largest number of annotated hits, with 61,483 (30.97%) and 69,549 (30.17%) unigenes for *A. chejuensis* and *A. quelpartensis*, respectively. This is not surprising, since the database contains only protostome protein sequences (Mollusca, Arthropoda and Nematoda). The database is more efficient than the NCBI non-redundant (nr) and Molluscs DB in terms of speed and quality of annotation [34]. The BLASTX annotation results for *A. chejuensis* and *A. quelpartensis* unigene sequences against PANM-DB are shown in Tables S2 and S3, respectively. We were able to map GO terms to 23,778 and 26,396 unigenes and KEGG pathways to 2246 and 2537 unigenes of *A. chejuensis* and *A. quelpartensis*, respectively. A size-based analysis of the BLAST annotated unigenes showed that longer unigenes were more likely to find matches in the reference public databases.

**Figure 3:**
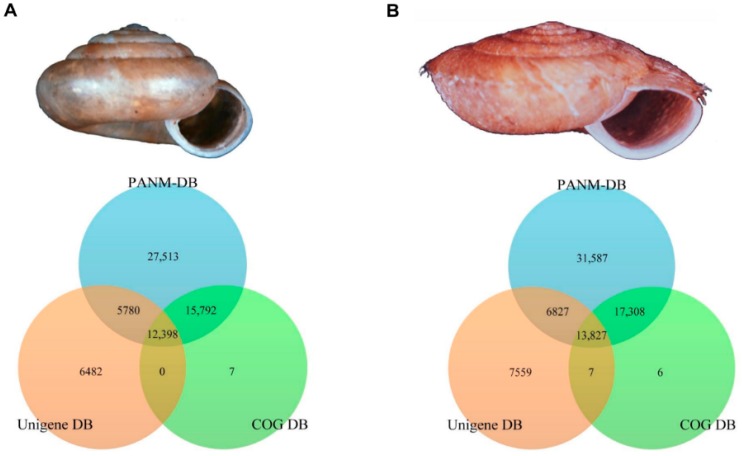
Sequence-based annotation of *A. chejuensis* (**A**); and *A. quelpartensis* (**B**) unigenes against PANM-DB, COG DB (BLASTX) and Unigene DB (BLASTN). The numbers represent the number of unigenes uniquely matched to homologous sequences in one, two or all three databases.

We also clarified the homologous matches of assembled unigene sequences in the PANM, Unigene, and COG databases using BLASTX with a cutoff *E*-value of 10^−5^ (Figure 3). Among the assembled unigenes annotated to the three public databases, 15,792 and 5780 unigenes in *A. chejuensis* had homologous sequences in PANM-DB and COG DB and PANM-DB and Unigene DB, respectively. A total of 12,398 unigenes were annotated by all three databases (Figure 3A). In the case of *A. quelpartensis* unigenes, 17,308 and 6827 unigenes were annotated concurrently by PANM-DB and COG DB, and by PANM-DB and Unigene DB, respectively. A total of 13,827 unigenes had homologous matches in all the three databases (Figure 3B). We found that most of the unigenes annotated to the functional COG DB also had homologous matches to protein sequences in PANM-DB. *A. quelpartensis* had more unigenes that showed homology to proteins in all three databases, which may reflect the larger number of transcripts available for this species. We also analyzed the assembled unigenes of both the species that annotated to all three databases (data not shown). The unigenes of *A. quelpartensis* and not *A. chejuensis* show homologous matches to G-type lysozyme and peroxiredoxin I in all three databases. Furthermore, homologous matches to both snail yolk ferritin and snail soma ferritin were noticed in the case of *A. quelpartensis* unigenes, while *A. chejuensis* unigenes found a homologous match to only snail soma ferritin. The annotated information for unigenes showing homologous matches in all three databases for both species includes catalase, β tubulin, importin β1, argonaute-2, kinesin light and heavy chain, troponin and tropomyosin, hedgehog, calmodulin, cathepsin, ubiquitin family, heat shock protein 70, C-type lectin, *etc.* These are specific examples and in no way represent the only results of the transcriptome annotation.

**Figure 4:**
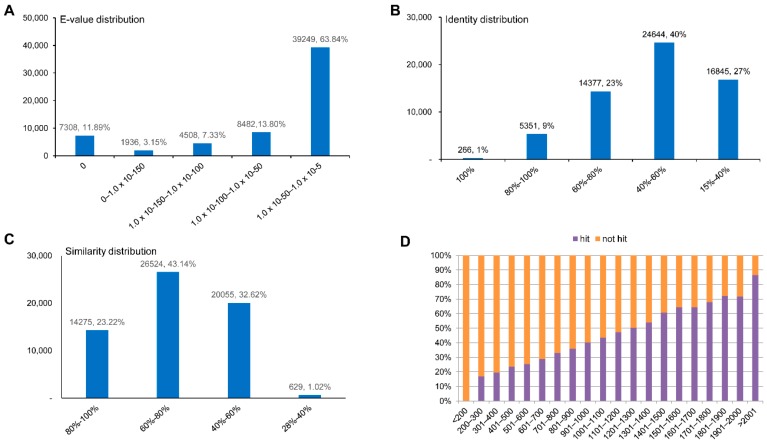
PANM-DB homology classification of *A. chejuensis* unigenes. (**A**) *E*-value distribution; (**B**) Identity distribution; (**C**) Similarity distribution; (**D**) Unigene hit or non-hit ratio.

We annotated unigenes using a BLAST search of PANM-DB, and assessed the *E*-values of alignments, sequence identity, similarity distribution, and the ratio of unigene hits to non-hits. In the case of *A. chejuensis* unigenes annotated to PANM-DB, the top hit *E*-value ranged from 1.0 × 10^−50^ to 1.0 × 10^−5^ (39,249 unigenes, 63.84%), followed by 1.0 × 10^−100^ to 1.0 × 10^−50^ (8482 unigenes, 13.8%), and 0 (7308 unigenes, 11.89%) (Figure 4A). We found that 40% of the mapped sequences had identities between 40%‒60% to matches in PANM-DB, while 23% of sequences had identities of 60%–80% (Figure 4B). Regarding the similarity distribution of annotated unigenes, most sequences (66.36%) had similarity higher than 60%, and 23.22% of sequences had similarity higher than 80% (Figure 4C). The hit percentage of unigenes increased in direct proportion to the length of the annotated unigenes, with approximately 85% of sequences longer than 2001 bp having hits to matching proteins in PANM-DB (Figure 4D).

**Figure 5:**
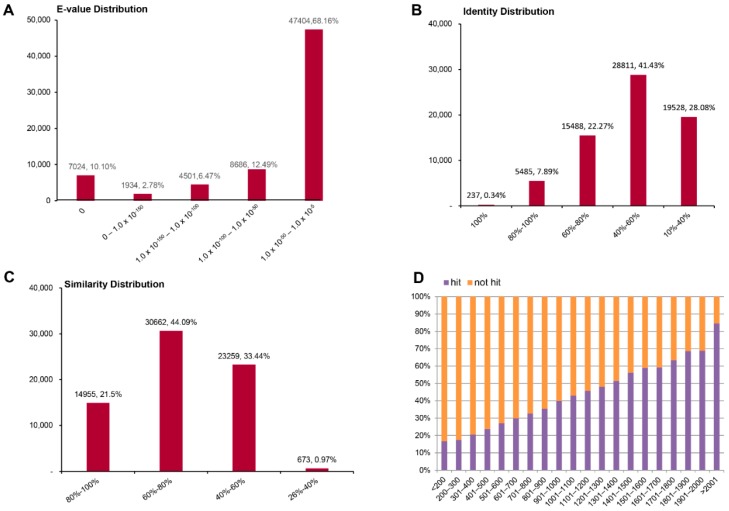
PANM-DB homology classification of *A. quelpartensis* unigenes. (**A**) *E*-value distribution; (**B**) Identity distribution; (**C**) Similarity distribution; (**D**) Unigene hit or non-hit ratio.

**Figure 6:**
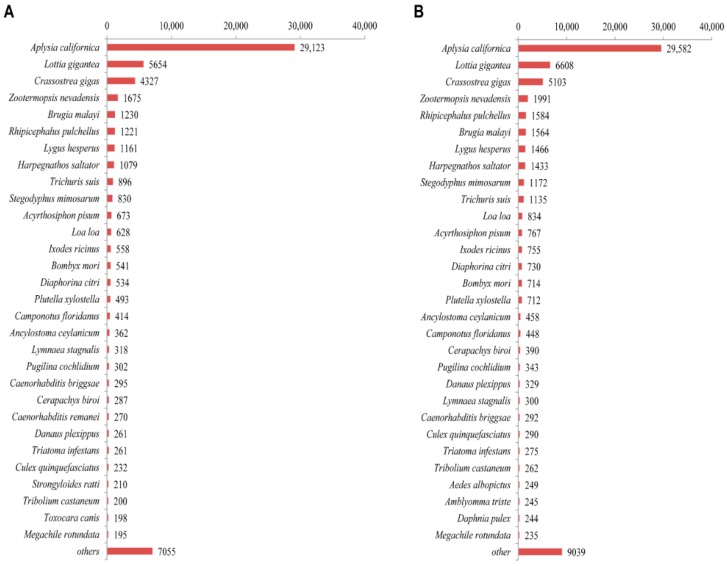
PANM-DB based top-hit species classification for (**A**) *A. chejuensis*; and (**B**) *A. quelpartensis* using BLASTX analysis.

The characteristics of the homology search for *A. quelpartensis* unigenes annotated against PANM-DB are shown in Figure 5. The *E*-value distribution of the top matches in PANM-DB showed that 68.16% of unigenes displayed evidence indicating a high degree of homology (1.0 × 10^−50^–1.0 × 10^−5^) to known genes, whereas 12.49% and 10.10% of sequences had *E*-values ranging from 1.0 × 10^−100^ to 1.0 × 10^−50^ and 0, respectively (Figure 5A). Forty-two percent of sequences showed identity of 40%–60%, and 22% of sequences showed identity of 60%–80% to matches in PANM-DB (Figure 5B). A large proportion of the unigenes (44.09%) showed similarity of 60%–80%, while 21.50% of unigenes showed similarity greater than 80% to the top annotated sequences in PANM-DB (Figure 5C). As with *A. chejuensis*, the number of annotated hits of *A. quelpartensis* unigenes increased with length; BLAST hits were obtained for approximately 85% of sequences longer than 2001 bp (Figure 5D). The species distribution showed that the greatest number of matches for *A. chejuensis* (47.37% of PANM-DB annotated unigenes) and *A. quelpartensis* unigenes (42.53% of PANM-DB annotated unigenes) were with *Aplysia californica* genes for which the genomic resources have previously been characterized in detail [35,36]. There were also top hit matches of unigene sequences to known proteins of the marine gastropod mollusc *Lottia gigantea* and the Pacific oyster *Crassostrea gigas*. The top hit species distributions for *A. chejuensis* and *A. quelpartensis* are shown in Figure 6A,B, respectively.

### 2.3. Functional Prediction Using COG, GO and KEGG

**Figure 7:**
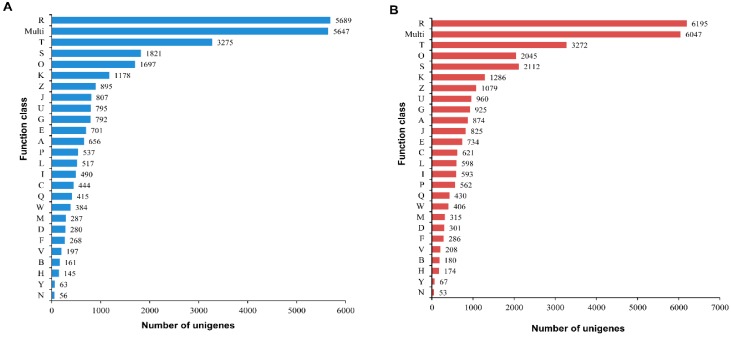
COG classification of (**A**) *A. chejuensis* and (**B**) *A. quelpartensis* unigenes. The code descriptions for COG categories are as follows: R, general function prediction only; Multi, more than one classified function; T, signal transduction mechanisms; S, unknown function; O, post-translational modification, protein turnover, and chaperones; K, transcription; Z, cytoskeleton; J, translation, ribosomal structure, and biogenesis; U, intracellular trafficking, secretion, and vesicular transport; G, carbohydrate transport and metabolism; E, amino acid transport and metabolism; A, RNA processing and modification; P, inorganic ion transport and metabolism; L, replication, recombination, and repair; I, lipid transport and metabolism; C, energy production and conversion; Q, secondary metabolites biosynthesis, transport and catabolism; W, extracellular structures; M, cell wall/membrane/envelope biogenesis; D, cell cycle control, cell division, and chromosome portioning; F, nucleotide transport and metabolism; V, defense mechanisms; B, chromatin structure and dynamics; H, co-enzyme transport and metabolism; Y, nuclear structure; N, cell motility.

The assembled *A. chejuensis* and *A. quelpartensis* unigenes were annotated against the proteins in the COG database. As a platform, the COG analysis classifies gene products into atleast 25 protein families broadly categorized to “metabolism”, “cellular processes and signaling”, “information storage and processing” or “poorly characterized groups”. In this study, a total of 28,197 and 31,148 unigenes from *A. chejuensis* and *A. quelpartensis*, respectively, were classified into 25 functional categories (excluding the multi-class category) (Figure 7). For both species, a large proportion of annotated sequences fell into the “general function prediction only” and “signal transduction mechanisms” categories, as well as the multi-class category. For *A. chejuensis* and *A. quelpartensis* sequences annotated against the COG database, 20.18% and 19.89% of unigenes, respectively, were grouped into “general function prediction only” category. The groups to which the fewest unigenes were annotated in both species included “defense mechanisms”, “chromatin structure and dynamics”, “coenzyme transport and metabolism”, “nuclear structure”, and “cell motility”. The COG-based functional predictions of annotated *A. chejuensis* and *A. quelpartensis* unigenes are shown in Figure 7A,B, respectively. Our results are consistent with the COG-based functional prediction of unigenes in the Littorinid snail *Echinolittorina malaccana* [15], invasive golden apple snail *Pomacea canaliculata,* and the mud snail *Cipangopaludina cahayensis* [37].

**Figure 8:**
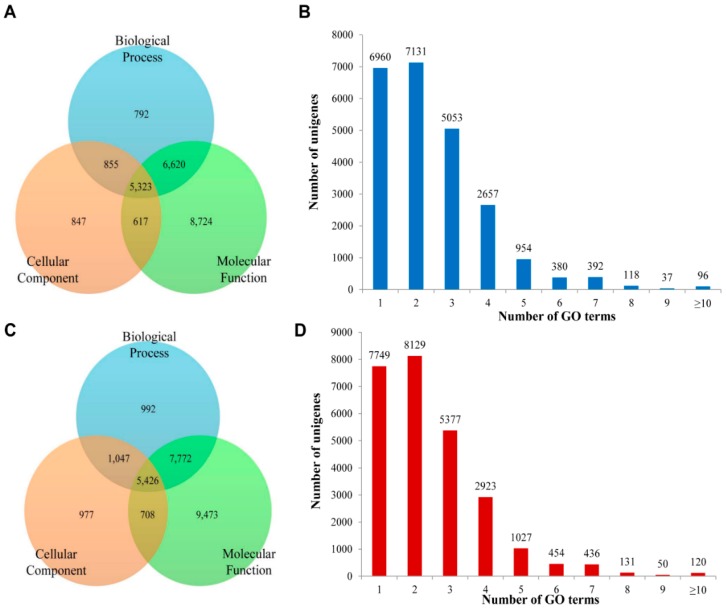
The functional prediction of unigenes under GO classification. (**A**) The distribution of the unigenes of *A. chejuensis* to GO biological function, cellular component and molecular function; (**B**) The number of GO terms ascribed to unigenes of *A. chejuensis*; (**C**) The GO functional prediction of *A. quelpartensis* unigenes; (**D**) The number of GO terms ascribed to unigenes of *A. quelpartensis*.

Functional analysis with the BLAST2GO suite was conducted on *A. chejuensis* and *A. quelpartensis* unigenes to identify associated GO terms and KEGG pathways. GO classification matches a gene to others of known (or predicted) function, but does not provide conclusive evidence of its function. The three main GO terms were “biological process”, “molecular function”, and “cellular component”. Among the 23,778 unigenes annotated for *A. chejuensis*, 8724, 847, and 792 sequences were classified into “molecular function”, “cellular component”, and “biological process”, respectively (Figure 8A). A total of 6620 unigenes were classified into both the “molecular function” and “biological process” categories, while 5323 sequences were classified into all three categories of GO term annotations. Moreover, 6960 (29.27%) unigene sequences annotated with a single GO term, and the remaining 16,818 (70.73%) sequences were annotated with more than one GO term (Figure 8B). Of the 26,396 unigenes annotated for *A. quelpartensis*, 9473, 992, and 977 sequences were assigned to the “molecular function”, “biological process”, and “cellular component” categories, respectively (Figure 8C). In addition, 5426 unigenes were classified into all three categories. A total of 7749 (29.36%) unigenes were annotated with a single GO term, while the remaining unigenes (18,647, 70.64%) were annotated with more than one GO term (Figure 8D).

**Figure 9:**
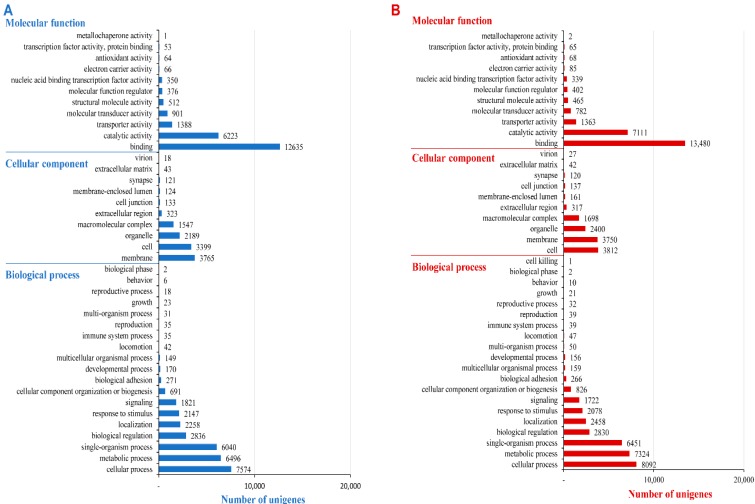
GO functional classification at level 2 for *A. chejuensis* (**A**); and *A. quelpartensis* (**B**) unigenes.

The top represented GO terms under the three functional categories (on level 2) for *A. chejuensis* are shown in Figure 9A. Within the category of “biological process”, most GO terms were grouped into “cellular process” (GO: 0009987), “metabolic process” (GO: 0008152) and “single-organism process” (GO: 0044699). Within the ‘cellular component’ category, the most highly represented GO terms were “membrane” (GO: 0016020), “cell” (GO: 0005623), and “organelle” (GO: 0043226). Among the 11 “molecular function” subcategories, most unigenes were assigned to “binding” (GO: 0005488), followed by “catalytic activity” (GO: 0003824) and “transporter activity” (GO: 0005215). GO term annotations for unigenes in the categories “response to stimulus” (GO: 0050896), “signaling” (GO: 0023052), and “immune system process” (GO: 0002376) helped to shed light on the adaptations of such endemic land snails. The assignment of GO functional terms to predicted *A. quelpartensis* unigenes followed a similar trend. The assigned sequences were categorized into 40 subcategories within three main categories, including “biological process” (19), “molecular function” (11), and “cellular component” (10) (Figure 9B). Within the “biological process” category, “cellular process”, “metabolic process” and “single-organism process” represented the most common GO terms. Within the “molecular function” category, “binding” and “catalytic activity” were the main GO assignments; within the “cellular components” category, the main assignments were “cell”, “membrane”, “organelle” and “macromolecular complex” (GO: 0032991). GO-based annotation suggested a diverse functional categorization of the predicted unigenes in *A. chejuensis* and *A. quelpartensis*, which is consistent with the results from the sequenced land snail *T. pisana* [20] and the freshwater snail *O. hupensis* [18]. In inferring functionality from GO terms, it is important to emphasize that not all GO terms are of equal validity, and that unigene function can be only predicted, not determined with certainty [38]. A GO classification, therefore, is not conclusive evidence of functionality and, in the absence of experimental verification, GO results can only suggest functionality. A large number of false positives are likely in many species as the majority of GO terms are assigned the code “IEA” (inferred from electronic annotation). These are terms that have not been manually curated and hence are of questionable validity. The GO term annotation in this study hence suggests that a gene is grouped to those of known (or predicted) function.

**Figure 10:**
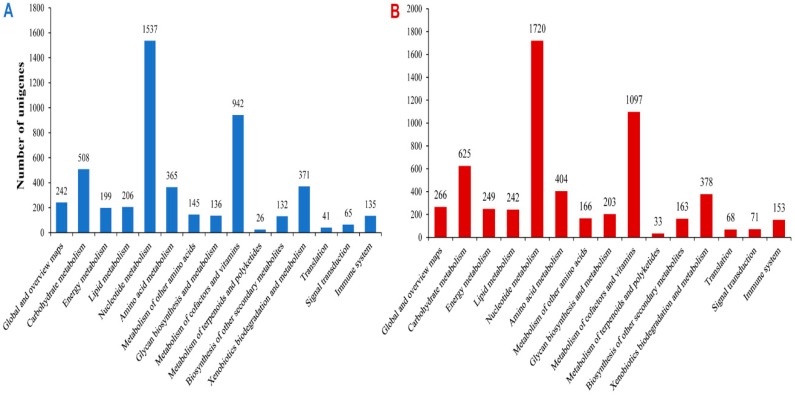
KEGG classification of unigenes in *A. chejuensis* (**A**); and *A. quelpartensis* (**B**).

In addition to COG analysis and GO annotation, we mapped the *A. chejuensis* and *A. quelpartensis* predicted proteins to reference KEGG pathways for functional categorization and annotation (Figure 10). Annotation based on the KEGG database is an alternative method to assess the involvement of unigenes in significant biological pathways, thus providing insight into intracellular metabolic functions. The assembled unigenes fell into KEGG pathways corresponding to “metabolism”, “genetic information processing”, “environmental information processing”, and “organismal systems”. For *A. chejuensis,* this process led to the assignment of 5050 assembled unigene sequences to a total of 117 pathways, while 5838 sequences were assigned to 124 pathways for *A. quelpartensis*. The unigenes identified from *A. quelpartensis,* but not *A. chejuensis,* relates to caffeine metabolism; d-alanine, d-glutamine, and d-glutamate metabolism; flavone and flavonol biosynthesis; lysine biosynthesis; and other types of *O*-glycan biosynthesis pathways. Metabolic pathways were highly represented with 4809 and 5546 unigenes for *A. chejuensis* and *A. quelpartensis*, respectively, associated with basic metabolism functions. Among the “signal transduction mechanism” pathways, unigenes from both species were assigned to “mTOR signaling pathway” and “phosphatidylinositol signaling system”. Unigenes from both species were assigned to the immune system “T-cell receptor signaling pathway”. The KEGG pathway resources for land snails of the genus *Aegista* may help reveal the specific bioprocesses related to its successful adaptation to a particular habitat, as well as possible gene plasticity allowing survival in other environments.

### 2.4. Protein Domain Identification Using InterProScan Searches

Using InterProScan, we annotated 6826 and 7170 unique protein domains among the assembled unigenes of *A. chejuensis* and *A. quelpartensis*, respectively. The most well-represented protein domains identified in *A. chejuensis* unigenes were the zinc finger, C2H2-like domain (IPR015880), G-protein-coupled receptor, rhodopsin-like family domain (IPR000276), and Ribonuclease H-like domain (IPR012337) (Table 3). Other domains with sequence hits included the ankyrin repeat (IPR002110), protein kinase (IPR002290), RNA recognition motif (IPR000504), WD40 repeat (IPR001680), immunoglobulin-like fold (IPR013783), and C-type lectin domain (IPR001304). The protein domains most highly represented based on *A. quelpartensis* unigene annotation are shown in Table 4. As with *A. chejuensis*, the dominant domains included the transcription factor zinc finger domains, protein kinase domains, and RNA recognition motif domains, which are pivotal in cellular regulatory processes.

The zinc finger C2H2-like domains are ubiquitous protein domains responsible for interaction with nucleic acids and protein targets [39]. Generally, multiple clusters of C2H2 zinc finger domains enable highly specific nucleic acid binding, thus assisting in cell fate determination and early developmental processes [40]. Recently, an expansion of C2H2-like zinc finger domains was identified in amphioxus and the California two-spot octopus, *Octopus bimaculoides* [41]. The C-type lectin, WD-40, protein kinase, catalytic, ankyrin repeat and immunoglobulin-like fold domains were also found to be abundant InterPro domains in the *Mytilus galloprovincialis* digestive gland transcriptome [28]. Protein kinase domains and WD40 repeat domains are conserved sequences involved in signal transduction functions and apoptosis [42,43]. Immunoglobulin-like fold repeat motifs are characteristic of proteins involved in the immune system and cellular processes, and are mediators of protein–protein interactions [44,45]. The InterProScan protein domain identification complements the functional annotation of unigenes, but is not error-free since it is based on electronic annotation. Hence, it could be considered as a preliminary step to unravel the putative functions of assembled sequences from transcriptome characterization.

### 2.5. Simple Sequence Repeat (SSR) Identification

SSRs are short sequences of 2–6 bases, and are established molecular markers in gene polymorphism studies and genomics applications. The SSRs in cDNAs are considered more transferable than random genomic SSRs because SSRs in genes are likely to be more conserved across taxa than SSRs from noncoding regions [46]. Therefore, the strength of transcriptome-derived SSRs in facilitating evolutionary analyses is due to the fact that they likely occur in the protein-coding regions of annotated unigenes [47,48,49]. These SSRs can be used to analyze the attributes of functional genes in association with their phenotypes [50]. We identified SSRs in 37,869 and 40,573 unigene sequences longer than 1 kb from *A. chejuensis* and *A. quelpartensis*, respectively. A total of 9542 and 10,395 SSRs comprising dinucleotide to hexanucleotide repeats were identified as high-priority markers. Mononucleotide microsatellites may arise from errors in sequencing homopolymeric regions, and thus were not considered for this study. We have provided a list of primer sequences that can be utilized to target the potential polymorphic SSRs in the two species (Tables S4 and S5 for *A. chejuensis* and *A. quelpartensis*, respectively). To characterize the markers in more detail and improve their utility for conservation genetics, we provide the PANM-DB annotation of SSR-containing sequences. Some of these microsatellite markers are located in unigene sequences related to innate immunity and defense (such as T-cell receptor, fibrinogen related protein, TRAF3, lectins, tumor necrosis factor *etc.*), defense processes against oxidative stress and other environmental perturbations (such as acetylcholine receptor, cytochrome P450 *etc.*), and regulatory binding/interaction processes (including the zinc finger motifs and serine/threonine motifs). This information may be useful for future work related to conservation genetics and population genetics for these two species endemic to Korea.

**Figure 11:**
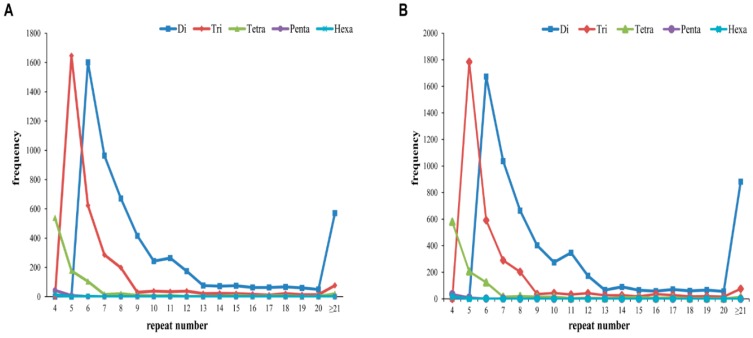
Summary of classified SSR repeat types in *A. chejuensis* (**A**); and *A. quelpartensis* (**B**) transcriptome.

In both species, dinucleotide repeat motifs were the most common type of marker, followed by trinucleotide and tetranucleotide motifs. In *A. chejuensis*, dinucleotide repeats with six tandem iterations, trinucleotide repeats with five tandem iterations, and tetranucleotide repeats with four tandem iterations were the most common (Figure 11A). Among the different numbers of tandem repeats, six tandem iterations were more common, followed by five and seven tandem iterations. The majority of pentanucleotide and hexanucleotide repeats showed a maximum of four tandem iterations. In *A. quelpartensis*, the frequencies of various types of SSRs were similar to those of *A. chejuensis*. The most frequent repeats were dinucleotides, which accounted for 57.58% of all SSRs, followed by trinucleotides (31.62%) and tetranucleotides (10.10%). Six iterations were most common among dinucleotide repeats, while five iterations were the most common among trinucleotide repeats. The less-common pentanucleotide and hexanucleotide repeats had a maximum of four iterations. SSRs with few tandem iterations were more common than those with many iterations, with the most common class being *n* = 6. A summary of the classified repeat types in *A. quelpartensis* unigenes is shown in Figure 11B.

**Figure 12:**
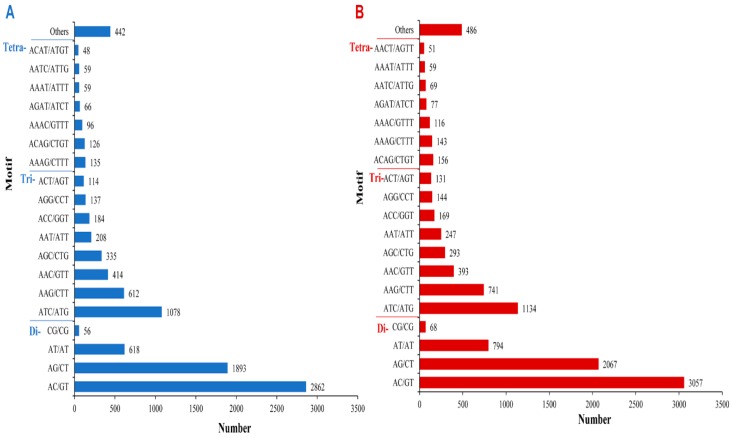
Frequency distribution of SSR repeat types in *A. chejuensis* (**A**); and *A. quelpartensis* (**B**) transcriptome.

We also identified the most common repeat motif types among SSRs (Figure 12). Among *A. chejuensis* SSRs, AC/GT motifs (29.99%) were most abundant among dinucleotide repeats, while ATC/ATG motifs (11.30%) and AAAG/CTTT (1.41%) were the most abundant among trinucleotide and tetranucleotide repeats, respectively. A detailed profile of the SSR motif types identified in *A. chejuensis* is shown in Figure 12A. Among the *A. quelpartensis* SSRs, AC/GT (29.41%) was also the most abundant dinucleotide repeat type (Figure 12B). The most abundant trinucleotide and tetranucleotide motifs were ATC/ATG (10.91%) and ACAG/CTGT (1.5%), respectively. In the invasive snail *P. canaliculata* [37] and oyster *Crassostrea hongko*ngensis [16], dinucleotide repeats with AG/CT and AT/AT repeat motifs were the most abundant SSRs. We also identified AT/AT repeat types in both *A. chejuensis* and *A. quelpartensis* SSRs. In the snail *E. malaccana*, the most common dinucleotide motif types in SSRs were AC/GT, followed by AG/CT [15]. SSRs identified from unigenes will facilitate future research on the genetic diversity and conservation of these species.

## 3. Experimental Section

### 3.1. Sample Preparation, cDNA Synthesis and Illumina Sequencing

*A. chejuensis* and *A. quelpartensis* were collected in living conditions from evergreen Jeju Jeolmul National Recreation Forest in Myeongnim-ro, Jeju-si, Jeju-do Island, Korea in August 2014. Our collection was permitted and assisted by the forest institute. Since these land snails are not on the list of endangered or protected species, no other permission was required. The snails were brought to the laboratory and rinsed in double distilled H_2_O to remove the mud and other particles attached to the shell. Snails were maintained at room temperature, within built-in enclosures and provided with water and food *ad libitum.* The snails were carefully removed from their shells with care to remove shell fragments. Whole-body samples of *A. chejuensis* and *A. quelpartensis* (*n* = 10) were frozen in liquid nitrogen for RNA extraction.

Total RNA was extracted from the snap-frozen homogenized samples using TRIzol Reagent (Invitrogen, Carlsbad, CA, USA) following the manufacturer’s protocol. RNA samples were treated with RNase-Free DNase I to eliminate genomic DNA. The purity and integrity of the extracted RNA was confirmed using the NanoDrop-2000 Spectrophotometer (Thermo Scientific, Wilmington, DE, USA) and Agilent 2100 Bioanalyzer with a minimum integrity number value of 7.

Oligo(dT) beads were used to elute poly(A) mRNA after RNA extraction. The mRNA was fragmented using fragmentation buffer to obtain short fragments prior to cDNA synthesis. First-strand cDNA synthesis was carried out using random-hexamer primers, with the short fragments as templates. Second-strand cDNA synthesis was carried out using buffer, dNTPs, RNaseH, and DNA polymerase I. The synthesized double-stranded cDNA was purified using the QiaQuick PCR extraction kit (Qiagen Inc., Valencia, CA, USA) and resolved with EB buffer for end-repair and A-tailing. Subsequently, sequencing adapters were attached to the fragments. The fragments were purified by agarose gel electrophoresis and enriched by PCR amplification. The cDNA library was sequenced by GnC Bio Company, Daejeon, Korea, using the Illumina HiSeq 2500, according to the manufacturer’s instructions. The transcriptome datasets of *A. chejuensis* and *A. quelpartensis* are available from the NCBI Sequence Read Archive (SRA) under the accession numbers SRP064881 (Project number PRJNA298949) and SRP064882 (Project number PRJNA298950), respectively. The datasets with the assembled contig information can be downloaded on or after 16 October 2016 (release date) [51].

### 3.2. De novo Transcriptome Assembly

The raw reads generated by Illumina sequencing were transformed by base calling and preprocessed to remove adapter fragments, ambiguous reads (*i.e.*, reads more than 5% unknown nucleotides) and low-quality sequences (Phred quality score <20 bases). Overlapping high-quality reads were used to create longer contiguous fragments (contigs) using the Trinity short reads assembler [52]. Assembly was carried out using the default Trinity options and a minimum length of 200 nt. The contig N50 value was computed using the Trinity script: % $TRINITY_HOME/util/TrinityStats.pl Trinity.fasta. Next, we used the TIGR Gene Indices Clustering Tools (TGICL) [53] a sequence clustering software, to cluster contigs into unigenes. The unigene sequences constitute expressed assembled sequences, but are not characterized sufficiently to be represented as a gene. For TGICL, we used the default parameters: *n* (number of sequences in a clustering search slice)—1000, *p* (minimum percent identity for overlaps)—94, I (minimum overlap length)—30, and v (maximum length of unmatched overhangs)—90.

### 3.3. Functional Annotation

The assembled unigenes profile was determined by a sequence-based annotation against Protostome database (PANM-DB) using BLASTX alignment (*E*-value ≤ 1 × 10^−5^). PANM-DB is a database for the analysis of molluscan NGS data, and contains protein sequences from Arthropoda, Nematoda, and Mollusca [34]. PANM-DB is linked to the amino acid BLAST web-interface of the Malacological Society of Korea [54]. Additional databases used for unigene annotation included the Unigene (BLASTN; *E*-value ≤ 1 × 10^−5^), Clusters of Orthologous Groups (COG), and Gene Ontology (GO) DB. The best-aligned results were used to determine the sequence direction and coding sequence (CDS) of the unigenes. GO and Kyoto Encyclopedia of Genes and Genomes (KEGG) annotations were assigned using the Blast2GO program [55]. WEGO software [56] was used to suggest the GO functional classification. The protein domain hits for assembled unigenes were recorded based on an InterProScan search in the Blast2GO interface [57].

### 3.4. SSR Motifs Detection

Simple sequence motifs (SSR) were identified using the program MicroSAtellite (MISA) [58] on unigenes of *A. chejuensis* and *A. quelpartensis* longer than 1 kb. The parameters defined for SSR analysis were as follows: di-, tri-, tetra-, penta-, and hexa-nucleotide repeats with a minimum of six, five, four, four, and four repeat numbers, respectively. Owing to the possibility of homopolymer generation during Illumina sequencing, we excluded mononucleotide repeats from our analysis. The BatchPrimer 3 program [59] was used to design the primers flanking the SSR motifs for polymorphism analysis. Primers were designed based on the following criteria: dinucleotides with six or more iterations and tri-/tetranucleotides with a minimum of four iterations. The primer characteristics were as follows: primer length 18–23 bases with an optimum size of 21 bases, product size of 100–300 bases, Tm (melting temperature) ranging from 50 to 70 °C, and primer GC% of 30%–70%. We also predicted unigene function based on homology to PANM-DB sequences (*E*-value cut-off of 10^−5^).

## 4. Conclusions

Our study represents the first transcriptome analysis for the land snails *A. chejuensis* and *A. quelpartensis,* which are endemic to Korea and are listed in the Red List of Threatened Species. Our results include novel genetic resources which may prove valuable in future research on the adaptive physiology and phylogeography of these species. Our genomic data were generated using Illumina HiSeq 2500 *de novo* transcriptome assembly, and functions were predicted using BLAST searches against public databases. Furthermore, SSRs identified from unigenes will facilitate the assessment of genetic diversity and conservation of these species in their natural habitat.

## Figures and Tables

**Table 1:** Statistics of transcriptome sequencing and assembling for *A. chejuensis* and *A. quelpartensis.*

Statistics	*A. chejuensis*	*A. quelpartensis*
Raw Reads		
Number of sequences	256,655,870	239,242,058
Number of total nucleotides	32,338,639,620	30,144,499,308
Clean Reads		
Number of sequences	253,220,985	235,525,993
Number of total nucleotides	31,397,895,789	29,188,212,057
Mean length (bp)	124	123.9
High-quality reads (%)	98.66 (sequences); 97.09 (bases)	98.45 (sequences); 96.83 (bases)
Number of reads discarded (%)	1.34 (sequences); 2.91 (bases)	1.55 (sequences); 3.17 (bases)
Assembled contigs		
Number of contigs	375,118	463,438
Number of total nucleotides	229,108,084	269,776,350
Mean length (bp)	610.8	582.1
N50 length (bp)	788	719
GC% of contig	42.02	41.53
Largest contig (bp)	34,543	26,467
Number of contigs ≥500 bp	124,882	145,244
Assembled unigenes		
Number of unigenes	198,531	230,497
Number of total nucleotides	145,998,300	162,627,732
Mean length (bp)	735.4	705.6
N50 length (bp)	1073	1001
GC% of unigene	41.98	41.40
Length ranges (bp)	105–34,543	100–29,273

**Table 2:** Annotation of *A. chejuensis* and *A. quelpartensis* unigenes against the public databases.

Databases	All Annotated Unigenes		≤300 bp		300–1000 bp		≥1000 bp	
	*A. chejuensis*	*A. quelpartensis*	*A. chejuensis*	*A. quelpartensis*	*A. chejuensis*	*A. quelpartensis*	*A. chejuensis*	*A. quelpartensis*
PANM	61,483	69,549	8882	11,191	27,318	32,401	25,283	25,957
Unigene	24,660	28,220	3857	4564	10,543	12,971	10,260	10,685
COG	28,197	31,148	2443	3085	8859	11,297	16,895	16,766
GO	23,778	26,396	2747	3467	8670	10,734	12,361	12,195
KEGG	2246	2537	190	230	634	877	1422	1430
ALL	68,484	77,745	10,515	13,037	31,552	37,410	26,417	27,298

**Table 3:** Top hit InterPro terms from the InterProScan annotations of *A. chejuensis* unigenes.

Domain	Description	Number of Unigenes
IPR015880	Zinc finger, C2H2-like domain	1655
IPR000276	G protein-coupled receptor, rhodopsin-like family	338
IPR012337	Ribonuclease H-like domain	322
IPR002110	Ankyrin repeat	307
IPR000477	Reverse transcriptase domain	299
IPR013087	Zinc finger C2H2-type/integrase DNA-binding domain	290
IPR027417	P-loop containing nucleoside triphosphate hydrolase domain	254
IPR002290	Serine/threonine/dual specificity protein kinase, catalytic domain	238
IPR003591	Leucine-rich repeat, typical subtype repeat	174
IPR002126	Cadherin domain	171
IPR000504	RNA recognition motif domain	169
IPR001680	WD40 repeat	164
IPR000742	EGF-like domain	162
IPR002048	EF-hand domain	154
IPR005135	Endonuclease/exonuclease/phosphatase domain	138
IPR013783	Immunoglobulin-like fold domain	136
IPR011701	Major facilitator superfamily	130
IPR002035	von Willebrand factor, type A domain	124
IPR012336	Thioredoxin-like fold domain	124
IPR001304	C-type lectin domain	118
IPR001478	PDZ domain	114
IPR019734	Tetratricopeptide repeat	114
IPR001841	Zinc finger, RING-type domain	111
IPR001245	Serine-threonine/tyrosine-protein kinase catalytic domain	104
IPR001849	Pleckstrin homology domain	103
IPR002172	Low-density lipoprotein (LDL) receptor class A repeat	103
IPR003593	AAA+ ATPase domain	103
IPR001452	SH3 domain	100
IPR003599	Immunoglobulin subtype domain	100
IPR000008	C2 domain	96
IPR011989	Armadillo-like helical domain	92
IPR007087	Zinc finger, C2H2 domain	90
IPR001888	Transposase, type 1 family	86
IPR000859	CUB domain	83
IPR001881	EGF-like calcium-binding domain	82
IPR003961	Fibronectin type III domain	80
IPR019427	7TM GPCR, serpentine receptor class w (Srw) family	79
IPR020846	Major facilitator superfamily domain	77
IPR002557	Chitin binding domain	75

**Table 4:** Top hit InterPro terms from the InterProScan annotations of *A. quelpartensis* unigenes.

Domain	Description	Number of Unigenes
IPR015880	Zinc finger, C2H2-like domain	1658
IPR000477	Reverse transcriptase domain	378
IPR012337	Ribonuclease H-like domain	360
IPR027417	P-loop containing nucleoside triphosphate hydrolase domain	322
IPR002110	Ankyrin repeat	322
IPR000276	G protein-coupled receptor, rhodopsin-like family	280
IPR013087	Zinc finger C2H2-type/integrase DNA-binding domain	249
IPR000504	RNA recognition motif domain	223
IPR002290	Serine/threonine/dual specificity protein kinase, catalytic domain	217
IPR001680	WD40 repeat	198
IPR002048	EF-hand domain	179
IPR005135	Endonuclease/exonuclease/phosphatase domain	176
IPR013783	Immunoglobulin-like fold domain	175
IPR003591	Leucine-rich repeat, typical subtype repeat	165
IPR002126	Cadherin domain	153
IPR000742	EGF-like domain	146
IPR011701	Major facilitator superfamily	145
IPR012336	Thioredoxin-like fold domain	140
IPR002035	von Willebrand factor, type A domain	126
IPR011989	Armadillo-like helical domain	116
IPR001841	Zinc finger, RING-type domain	109
IPR001888	Transposase, type 1 family	105
IPR019734	Tetratricopeptide repeat	104
IPR001304	C-type lectin domain	104
IPR001245	Serine-threonine/tyrosine-protein kinase catalytic domain	102
IPR016040	NAD(P)-binding domain	101
IPR001478	PDZ domain	101
IPR002347	Glucose/ribitol dehydrogenase family	100
IPR000008	C2 domain	98
IPR003593	AAA+ ATPase domain	97
IPR020846	Major facilitator superfamily domain	96
IPR001849	Pleckstrin homology domain	93
IPR029058	Alpha/Beta hydrolase fold domain	92
IPR001452	SH3 domain	92
IPR003599	Immunoglobulin subtype domain	90
IPR015943	WD40/YVTN repeat-like-containing domain	89
IPR007087	Zinc finger, C2H2 domain	89
IPR002172	Low-density lipoprotein (LDL) receptor class A repeat	86
IPR000859	CUB domain	86
IPR000719	Protein kinase domain	81

## Supplementary Materials

Supplementary materials can be found at http://www.mdpi.com/1422-0067/17/3/379/s1.
