# Supplementary material for: De novo Transcriptome Generation and Annotation for Two Korean Endemic Land Snails, Aegista chejuensis and Aegista quelpartensis, Using Illumina Paired-End Sequencing Technology
Source: Int J Mol Sci. 2016 Mar 15;17(3):379. doi: 10.3390/ijms17030379 (PMC4813237; doi:10.3390/ijms17030379)
Supplement: Supplementary file 1 [file ijms-17-00379-s001.zip › Supplementary Table S1.docx]

Supplementary Materials: *De novo* Transcriptome Generation and Annotation for Two Korean Endemic Land Snails, *Aegista chejuensis* and *Aegista quelpartensis*, Using Illumina Paired-End Sequencing Technology

Se Won Kang, Bharat Bhusan Patnaik, Hee-Ju Hwang, So Young Park, Tae Hun Wang, Eun Bi Park, Jong Min Chung, Dae Kwon Song, Hongray Howrelia Patnaik, Jae Bong Lee, Changmu Kim, Soonok Kim, Hong Seog Park, Jun Sang Lee, Yeon Soo Han and Yong Seok Lee

**Table S1.** Processing of raw reads after Illumina HiSeq 2500 sequencing of Korea endemic land snails, *A. chejuensis* and *A. quelpartensis*. The thinning and trimming analyses while processing of raw reads was done with Cutadapt program.

| **Data Processing Parameters** | ***Aegista chejuensis*** | ***Aegista quelpartensis*** |
| --- | --- | --- |
| Raw reads |  |  |
| Number of sequences | 256,655,870 | 239,242,058 |
| Number of bases | 32,338,639,620 | 30,144,499,308 |
| Total read pairs processed | 128,327,935 | 119,621,029 |
| Read 1 with adapter | 4,568,630 (3.6%) | 4,690,011 (3.9%) |
| Read 2 with adapter | 5,653,389 (4.4%) | 5,800,573 (4.8%) |
| Pairs written (passing filters) | 128,327,935 | 119,621,029 |
| Total base pairs processed (bp) | 32,338,639,620 | 30,144,499,308 |
| Read 1 (bp) | 16,169,319,810 | 15,072,249,654 |
| Read 2 (bp) | 16,169,319,810 | 15,072,249,654 |
| Total written (filtered) (bp) | 32,284,657,766 | 30,081,242,627 |
| Read 1 (bp) | 16,143,957,105 | 15,042,310,920 |
| Read 2 (bp) | 16,140,700,661 | 15,038,931,707 |
| % of reads after trimming | 99.83 | 99.79 |
| % of reads discarded | 0.17 | 0.21 |
| average length after trimming (bp) | 125.8 | 125.7 |
| Adapter 1 sequence | AGATCGGAAGAGCACACGTCTGAACTCCAGTCAC | |
| Adapter 2 sequence | AGATCGGAAGAGCGTCGTGTAGGGAAAGAGTGTA GATCTCGGTGGTCGCCGTATCATT | |
